# First Insights on the Upcoming Role of Next-Generation PLLA-LASYNPRO in Aesthetic and Regenerative Medicine: A Survey of Experts—the PLLA-LASYNPRO Rationale

**DOI:** 10.1055/a-2764-3062

**Published:** 2026-02-27

**Authors:** Dario Bertossi, Maurizio Cavallini, Alessandra Camporese, Roberto Dell'Avanzato, Nicola Kefalas, Enrico Massidda, Marco Papagni, Mariagrazia Patalano, Sandro Quartucci, Monica Renga, Adriano Santorelli, Chantal Sciuto, Gloria Trocchi

**Affiliations:** 1 Department of Surgical Sciences, Section of Oral and Maxillofacial Surgery, University of Verona, Verona, Italy; 2 Executive Committee of Agorà, Italian Society of Aesthetic Medicine, Milan, Italy; 3 International School of Aesthetic Medicine, Fondazione Internazionale Fatebenefratelli, Rome, Italy; 4 Private Practice, Milan, Italy; 5 Rigenera Lab, Turin, Italy; 6 Università Niccolò Cusano, Rome, Italy; 7 Private Practice, Studio Mariagrazia Patalano, Messina, Italy; 8 Saba Clinic, Rome, Italy; 9 Adriano Santorelli & Partners Private Practice, Naples, Italy; 10 Pathology Isola Tiberina Hospital - Gemelli Isola, Rome, Italy

**Keywords:** earlier-generation poly-L-lactic acid, extracellular matrix regeneration, foreign body response, injectable collagen stimulators, expert board, PLLA-LASYNPRO, skin quality

## Abstract

**Introduction:**

Injectable collagen stimulators have traditionally been linked to inflammatory foreign-body reactions (FBR) as a mechanism of action. However, the next-generation PLLA-LASYNPRO microspheres, contained in the CE-marked JULÄINE medical device, may represent a paradigm shift. Preclinical data suggest these microspheres can promote collagen and extracellular matrix (ECM) regeneration with minimal inflammatory response.

**Objectives and Hypotheses:**

This study aimed to evaluate the scientific soundness and clinical relevance of a non-inflammatory mechanism of action for PLLA-LASYNPRO. The central hypothesis was that design and manufacturing innovations could enable effective biostimulation while reducing inflammation and long-term tissue reactions.

**Study Design:**

A structured expert board meeting was convened to assess the rationale and implications of this emerging mechanism. The process included a preliminary survey and an in-person consensus meeting involving multidisciplinary specialists in aesthetic and regenerative medicine.

**Methods:**

On January 24, 2025, 13 experts in aesthetic medicine, dermatology, and plastic surgery participated in a board meeting held in Milan, Italy. Scientific literature and preclinical data were reviewed in advance. Discussions were organized around biophysical characteristics, tissue integration, inflammatory profile, and safety considerations.

**Results:**

The board considered the non-inflammatory mechanism of PLLA-LASYNPRO both biologically plausible and clinically promising. Key differentiating features included particle morphology, lack of excipients, and manufacturing purity. The panel highlighted the potential to reduce chronic inflammation, a known limitation of traditional collagen stimulators. Early clinical impressions supported this hypothesis, although prospective data are still forthcoming.

**Conclusion:**

This manuscript presents the consensus of a multidisciplinary board on the rationale for PLLA-LASYNPRO in aesthetic and regenerative medicine. It forms the first part of a two-paper series. The second manuscript will provide practical clinical guidance for the deep dermal administration of PLLA-LASYNPRO and real-world use of JULÄINE.

## Neosynthesis of Collagen and Extracellular Matrix: The Long-dominant Foreign Body Reaction Paradigm

Whether acknowledged or downplayed, the notion of an inflammatory foreign-body reaction (FBR) has been central to understanding how injectable resorbable collagen stimulators enhance the synthesis of collagen and other components of the extracellular matrix in connective tissues. The introduction of the first sterile, water-reconstituted poly-L-lactic acid (PLLA) formulation around the turn of the century marked the birth of the FBR concept. 1 2 3 The FBR is a spontaneous response that gradually encases and sequesters subdermal implants, such as traditional injectable collagen stimulators, in a fibrous shell. Unfortunately, inflammation also contributes to the most severe late side effects, including nodules and hardened skin indurations, which can occasionally progress into persistent granulomas lasting for months or even years. 1

A 2021 multicenter, retrospective chart review of U.S. medical records involving 4,483 treatments across the midface, temple, and jawline in 1,002 subjects revealed a persistently concerning long-term incidence of nodules at 0.4% for early-generation PLLA despite reconstituting the dry powder to 8 to 10 mL, a measure known and regulatory acknowledged to reduce long-term inflammatory adverse effects. 4 Other recent reviews indicate that the long-term incidence of late adverse effects—including nonvisible but palpable subcutaneous nodules, visible nodules, and chronic granuloma—ranges from a non-negligible 0.2 to 1.2%. 5 The burden of inflammatory late adverse effects appears unrelated to geographical latitude and ethnicity. Moreover, it is likely underestimated due to diagnostic challenges and the late occurrence, which masks the cause-and-effect relationship. 6 The injectable collagen bio-stimulator poly(ε-caprolactone) or PCL is not exempt from inflammatory side effects. However, inflammatory side effects occur only occasionally and are significantly milder, as confirmed by the U.S. FDA Center for Devices and Radiological Health. 2 3 7 According to a 2020 review of PCL, “The host response includes protein coating of the material, macrophage migration, and encapsulation at around three weeks. Inflammatory reactions and wound healing pathways participate in this stepwise repair process.” 8

Invoking the inflammatory FBR postulate seems less convincing for resorbable ceramic-derivative calcium hydroxylapatite. Unlike conventional PLLA microparticles, the macrophage expression of several pro-inflammatory cytokines, including IL-1α, IL- 1β, IL-8β, and chemokine (C-X-C motif) ligand 6 (CXCL6), appears significantly downregulated after exposure to the calcium hydroxylapatite microspheres. 9 10 However, other evidence seems to contradict these findings. Inflammatory biomarkers such as chemokine (C-X-C motif) ligand 8/IL-8 (CXCL8/IL-8), IL-6, and prostaglandin-endoperoxide synthase 2 (PTGS2) increase with calcium hydroxylapatite implants in over half of a panel of subjects treated for nasolabial folds. 11 After superficial subcutaneous injection compared with conventional PLLA, injected in the opposing arms of five female subjects, histology revealed a similar new production of collagen and elastic fibers. However, the moderate to intense inflammatory reaction involving lymphocytic and giant cell infiltrate was comparable. 12 More generally, the most recent literature does not exclude the evidence of focal accumulations and nodules with calcium hydroxylapatite. 13

## Is it Conceivable to Progress Beyond the Foreign Body Response in Skin Connective Tissue Regeneration?

The FBR postulate has never been denied or refused, although international literature has occasionally downplayed the inflammatory nature of induced extracellular matrix regeneration. A shift from the inflammatory FBR paradigm to a novel non-inflammatory collagen/EC regeneration that mitigate the risks of delayed inflammatory side effects may have emerged with the next-generation PLLA-LASYNPRO microspheres, usually injected subdermally using a sterile 25-gauge cannula. These uniform microspheres produced using advanced, patented freeze-drying technologies, do not disrupt the phagosome membranes, sparking FBR and causing the pro-inflammatory leakage of cathepsin into the cytosol. Their dermal regenerative action should develop independently from their phagocytosis and subsequent FBR. Furthermore, the smooth-surfaced, rounded PLLA microparticles preferentially activate the subpopulation of M2-polarized macrophages that secrete anti-inflammatory cytokines such as interleukin-4 (IL-4), IL-10, and IL-13. 14 15 Two decades after injectable earlier-generation PLLA received approval in Europe (1999) and the United States (2004), 16 a clinical program is underway in Europe to validate the non-inflammatory rationale behind the new PLLA-LASYNPRO functional ingredient and its anticipated efficacy and safety benefits as a significant turnaround. A recent Spanish interim multicenter analysis of 36 adult subjects confirmed the safety and rejuvenating efficacy of the PLLA-LASYNPRO microsphere implants on mild to severe nasolabial folds. 17 Assessed with two photo-numeric tools, the five-grade Wrinkle Severity Rating Scale (WSRS) and the six-point Midface Volume Deficit Scale (MFVDS) focused on middle third facial volume loss, 44.4 and 63.9% of subjects reported highly significant score improvements 1 and 2 months, respectively, after the first injection. 18 Wrinkle improvements were at least 1 point compared to baseline and uniform on the right and left facial sides. The circulating levels of procollagen type I carboxy-terminal propeptide (P1CP), a marker of type-1 collagen neosynthesis, rapidly showed significant increase 1 month after the first dose. The expected side effects, such as occasional edema, erythema, and infrequent local irritation, were mild, transient, and typical of all micro-invasive procedures. 17 Regarding the origin of this paper, a board of 13 experts in aesthetic and regenerative medicine, dermatology, and aesthetic plastic surgery convened to discuss and share their insights with their European colleagues about the rationale and role of PLLA-LASYNPRO subdermal implants. The focus of discussions was the available evidence and the author's direct clinical experience in the clinical research program currently being developed in Europe. Although research is progressing, the board experts believe their collaborative efforts deserve a broader audience, including several preliminary suggestions for integrating the novel CE-approved JULÄINE medical device based on PLLA-LASYNPRO subdermal implants into everyday regenerative medicine practice. The authors will incorporate such suggestions in the forthcoming manuscript.

## PLLA-LASYNPRO Rationale Beyond the FBR Paradigm

**Fig. 1:**
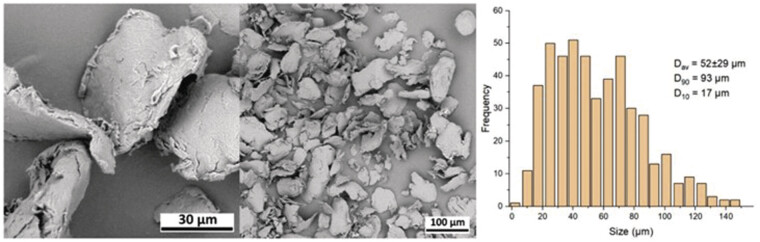
Scanning electron microscopy at two magnifications and size distribution of reconstituted early-generation, irregular, plate-like poly-L-lactic acid (PLLA) microparticles (first example of currently available formulation). Dav, average diameter; D10, D90, tenth and ninetieth diameter percentiles. Source: Republished from Sedush et al. 18 Licensed under CC BY 4.0.

**Fig. 2:**
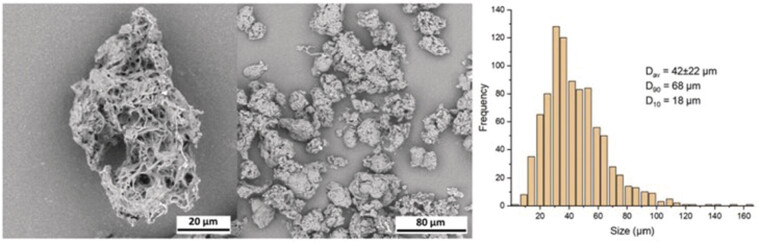
Scanning electron microscopy at two magnifications and size distribution of reconstituted early-generation, irregular, and highly porous poly-L-lactic acid (PLLA) microparticles (second example of currently available formulation). Dav, average diameter; D10, D90, tenth and ninetieth diameter percentiles. Source: Republished from Sedush et al. 18 Licensed under CC BY 4.0.

On average, early-generation PLLA microparticles appear oblong, irregular, and heterogeneous in size and shape. They resemble irregular, spiky micro-flakes ranging from 2 to 150 µm along their longer axis, with nearly half of the microparticles measuring less than 20 µm in diameter ( Figs. 1 and 2 ). 2 10 18 19 This characteristic makes early-generation PLLA microparticles susceptible to an inflammatory response and phagocytosis by macrophages, which can ultimately lead to the development of granulomas and delayed-onset nodules. Additionally, the microparticles in some formulations of early-generation PLLA are porous, further influencing inflammatory responses. 10 18 19 Furthermore, a sizable fraction of earlier PLLA microparticles at least 100 µm risk becoming trapped in the standard 26-gauge needle (internal diameter: 100 µm) used for injection. 18

**Fig. 3:**
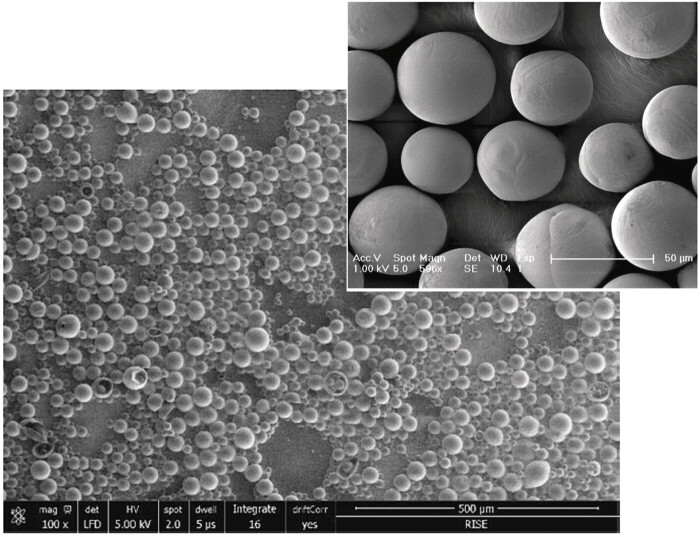
Scanning electron microscopy of PLLA-LASYNPRO microspheres at X100 (larger image) and X500 (upper-right detail) magnifications. 20 (Credit: Nordberg Medical SA (R&D), Huddinge, Sweden, with an agreement to publish.)

Conversely, the highly pure and readily dispersible microspheres of the innovative Class III JULÄINE medical device (Nordberg Medical AB, Huddinge, Sweden), produced using proprietary, patented freeze-drying technologies, exhibit precise spherical shapes and smooth surfaces with a non-porous structure. They possess a uniform diameter ranging from 20 to 50 µm (average: 33.3 µm) and exhibit a consistent in vivo degradation rate over 2 years, along with long-term stability and shelf life ( Fig. 3 ). 20

Each JULÄINE vial of dry powder contains 150 mg of PLLA-LASYNPRO microspheres; additional excipients include 45 mg of sodium carboxymethyl cellulose and 145 mg of non-pyrogenic mannitol, which do not appear to have a direct role in modulating inflammation. 20 Moisture and pro-inflammatory heavy metal and tin residues are below 0.5%, 0.001%, and 6.0 µg/mL (ppm), respectively, significantly lower than the levels found in earlier-generation PLLA derivatives. 20 The PLLA-LASYNPRO-based JULÄINE device has been available in Spain and Sweden for 2 years, and introduction proceeds in several Western European countries—France, Germany, Switzerland, Benelux, Portugal, the UK, and Italy.

## Non-inflammatory Action of the New-technology Microspheres

Even with earlier-generation PLLA formulations, the FBR paradigm does not fully explain collagen and extracellular matrix neosynthesis. For instance, the purely inflammatory FBR model does not clarify why a noticeable facial tightening effect often occurs just 1 month after earlier-generation PLLA injections, as the temporal framework seems too short. 21 In vitro evidence may provide a basis for elucidating the early tightening effects observed in vivo from earlier-generation PLLA formulations. For example, exposing fibroblasts to these conventional PLLA formulations for 48 hours activates the p38, Akt (protein kinase B), and JNK (c-Jun N-terminal kinase) signaling proteins, which are key regulators in signal transduction related to cell growth, differentiation, and apoptosis. This exposure also elevates the expression of the type-I collagen gene. 22 The Akt signaling pathway contributes to fibroblast migration, differentiation into myofibroblasts, collagen synthesis, and cutaneous wound contraction. Gene transcription increases rapidly within 48 hours and is likely independent of any FBR-like effects. Following the upregulation of type-I collagen gene expression, procollagen concentrations also rise quickly in the incubation medium. 22

Another well-known FBR-independent regenerative sequence of events that leads to collagen and ECM neosynthesis involves the pH-dependent activation of latent transforming growth factor-β (TGF-β). Acidic lactate, produced by the gradual degradation of PLLA, triggers the regenerative signal. 23 24 Furthermore, active TGF-β directs fibroblasts to adopt the contractile myocyte phenotype to differentiate into myofibroblasts, and enhance the production of extracellular collagen and matrix. Additionally, active TGF-β prompts fibroblasts and myofibroblasts to upregulate the lactate-generating enzyme LDHA (lactate dehydrogenase-A), resulting in persistently high lactate concentrations and continuous local TGF-β activation. 23 24 25 26 In vitro upregulation of the tissue inhibitor of metalloproteinase 1 (TIMP1) signaling pathway by lactic acid represents a third TGF-β-triggered event, ultimately leading to sustained inhibition of collagen catabolism. 27 28 All such non-inflammatory events appear strongly activated by PLLA-LASYNPRO subdermal implants. 21 Furthermore, the gradual degradation of the new- technology PLLA microspheres into lactate monomers supports cellular energy production through the tricarboxylic acid cycle and the electron transport chain. 29

**Fig. 4:**
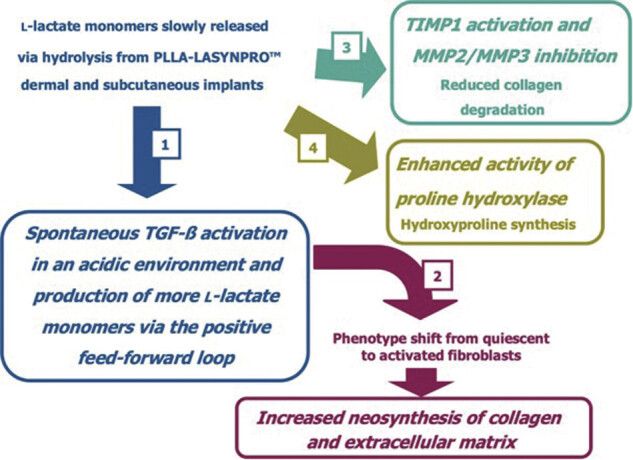
The non-inflammatory chain of events leading to neosynthesis of collagen and extracellular matrix: ( **1** ) Self-sustaining positive feed-forward loop of lactate-dependent TGF-β activation, sparked by the slow release of lactic acid monomers from PLLA-LASYNPRO implants and persistently supported over time by TGF-β-dependent lactate dehydrogenase (LDHA) upregulation (TGF-β: transforming growth factor-β). ( **2** ) TGF-β-dependent conversion of quiescent fibroblasts into actively collagen-producing contractile myofibroblasts. ( **3** ) Concomitant inhibition of extracellular collagen degradation by lactic acid monomers (TIMP1: tissue inhibitor of metalloproteinase 1). ( **4** ) The potential supportive role of lactic acid residues in stimulating proline hydroxylase leads to the expansion of the dermal and subcutaneous pool of the collagen-specific hydroxyproline amino acid. (Diagram drawn and owned by the authors based on information from Kottmann et al, 23 Judge et al, 24 Meng et al, 25 Vavřička et al, 29 Oh et al. 30 )

**Fig. 5:**
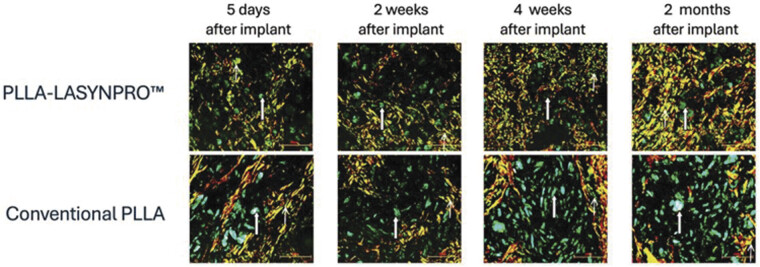
PLLA-LASYNPRO (upper row): Intense deposition of collagen (medium-thickness fibers shown in yellow; thick mature fibers shown in red) and extracellular matrix begins and increases after the 4th week around the dispersed subdermal new-technology microspheres (green staining and arrows), with no signs of aggregation. Conventional PLLA (commercial earlier-generation PLLA formulation): sparse and irregular neocollagenesis around persistent PLLA aggregates. Comparative evolution over the first 2 months (Picrosirius Red staining in lab models, X100 magnification). Scale bars (lower right corners) = 100 µm. 20 (Credit: Nordberg Medical SA (R&D), Huddinge, Sweden, with an agreement to publish.)

**Fig. 6:**
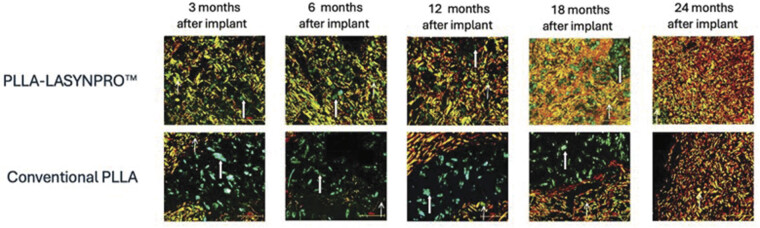
PLLA-LASYNPRO (upper row): increasingly dense and regular deposition of collagen and extracellular matrix (medium-thickness fibers shown in yellow; thick mature fibers shown in red), transitioning over 1 year to a progressive prevalence of definitive type-I fibers. The resorption of the PLLA-LASYNPRO microspheres (indicated by green staining and arrows) is nearly complete after 2 years, while the regular texture of newly deposited collagen fibers persists. Earlier-generation conventional PLLA: disorganized neocollagenesis at all times with slow degradation of PLLA microparticles and aggregates and, at 24 months, sparse surviving PLLA microparticles (arrow). The fibrous capsules surrounding hollow areas correspond to the resorbed earlier-generation PLLA microparticles. Comparative evolution from the 3rd month to 2 years (Picrosirius Red staining in lab models, X100 magnification). Scale bars (lower right corners) = 100 µm. 20 (Credit: Nordberg Medical SA (R&D), Huddinge, Sweden, with an agreement to publish.)

**Fig. 7:**
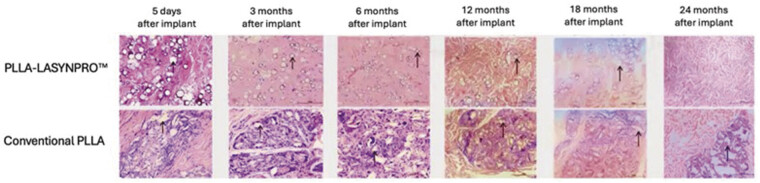
PLLA-LASYNPRO (upper row): Negligible inflammation over 24 months. The microspheres do not aggregate, remain detectable after 18 months, and wane entirely after 24 months, leaving no gaps in tissues that maintain their orderly structure. Conventional PLLA (commercial earlier-generation PLLA formulation): Irregular neocollagenesis with disruption of the orderly subdermal histology surrounding persistent PLLA aggregates. Gaps and tissue hollows are detectable after 18 and 24 months (hematoxylin and eosin staining in lab models, X100 magnification). Scale bars (lower right corners) = 100 µm. 13 20 (Credit: Nordberg Medical SA (R&D), Huddinge, Sweden, with an agreement to publish.)

Fig. 4 summarizes the likely in vivo effects on fibroblasts in dermal and subcutaneous connective tissues following exposure to the lactic acid monomers gradually released from the PLLA-LASYNPRO microspheres over several months. The activation of TGF-β by the acidic microenvironment induced by the lactate residues released from the microspheres promotes myofibroblast differentiation, collagen synthesis, and the formation of the extracellular matrix while reducing collagen degradation with minimal inflammatory responses ( Figs. 5 and 6 ). 2 19 23 24 25 30 The negligible inflammatory response, with no scar-like tissues or nodules and even dispersion without focal aggregations of the steadily and slowly degrading new-technology PLLA microspheres, persists for 24 months. There are no tissue compressions or deformities. Some microspheres remain detectable at month 18, with complete degradation occurring by month 24, leaving no residue or tissue gaps after degradation ( Fig. 7 ). 14 20 The positive feedback loop would wane with the resorption of the microspheres, thus eliminating any long-term risk of fibrosis. The stimulating role of lactate on fibroblast collagen proline hydroxylase may also promote self-sustaining neocollagenesis. 26 27 The increased levels of IL-4 and IL-13 led to macrophage polarization toward the M2 subtype and tissue remodeling, further enhancing TGF-β secretion. 30 Thanks to all previous considerations, the PLLA-LASYNPRO functional ingredient can be described as a long-acting, deep biostimulator or bioregenerator, not a filler. Moreover, recent in vitro studies with cultured adipocytes suggest that PLLA monomers may help stimulate adipogenesis in subcutaneous adipose tissues, potentially countering the loss of subcutaneous fat due to aging and photoaging, possibly contributing to deep wrinkles. 31 PLLA monomers are increasingly emerging as crucial signaling factors in the cell machinery. For instance, L-lactate protects mitochondria in skin fibroblasts from aging-related dysfunction. Mito-hormesis, the name of the modulation process, is a persistent cellular adaptive response of mitochondria and mitochondria-associated membranes to mild stressors whereby skin fibroblasts enhance their survival and stress resistance, possibly by inducing the release of stress-triggered mitokines fibroblast growth factor 21 (FGF21) and growth and differentiation factor 15 (GDF15). 32

## Conclusion

Technological innovations have led to the novel PLLA-LASYNPRO subdermal implants. Are these implants a genuine breakthrough in addressing the inflammatory foreign-body response paradigm? We know from the past that some degree of inflammation has consistently accompanied the action of resorbable collagen inducers before the new-technology PLLA derivative. 1 The Next-Generation PLLA-LASYNPRO™ Regenerative Medicine Expert Board cautiously endorsed the non-inflammatory rationale behind the new medical device. They found the initial findings from preclinical and microscopic investigations 2 11 20 21 22 23 24 25 26 27 28 29 30 33 persuasive and aligned with their clinical experience as leaders in the ongoing clinical research program of the new PLLA technology. The CE-approved JULÄINE medical device, based on novel PLLA technology, may effectively address the challenge of inflammatory side effects. Still, long-term evidence of efficacy and safety is lacking, and the authors acknowledge this is an undeniable bias that can be remedied only in the future. However, the no-inflammation PLLA-LASYNPRO rationale appears solid and not contradicted by initial clinical evidence. 17 32

The Next-Generation PLLA-LASYNPRO™ Regenerative Medicine Expert Board is confident that the new PLLA technology might emerge as a breakthrough in skin regeneration with no more than mild and transitory inflammation. Caution and further research efforts remain essential in this initial phase to substantiate the first favorable results.
